# Electrical experimental data collection of polycrystalline and monocrystalline photovoltaic modules in an indoor environment using artificial sun simulator

**DOI:** 10.1016/j.dib.2022.108389

**Published:** 2022-06-14

**Authors:** Ahmed Al Mansur, Md. Imamul Islam, Mohammad Kamrozzaman Kiron, Mohammad Asif ul Haq, Md. Hasan Maruf, ASM Shihavuddin, Ratil Hasnat Ashique, Md. Ruhul Amin

**Affiliations:** a Department of Electrical and Electronic Engineering, Green University of Bangladesh, Dhaka, Bangladesh; b Department of Computer Science and Engineering, Indipendent University of Bangladesh, Dhaka, Bangladesh; c Department of Electrical and Electronic Engineering, Islamic University of Technology, Gazipur, Dhaka, Bangladesh

**Keywords:** Photovoltaic characteristics, electrical data, PV performance, artificial sun simulator

## Abstract

In the twenty-first century, energy sustainability and reliability are one of the major challenges in the world and prime factors of the national development plan. Recently, Solar PV is gaining popularity and making a significant effect as an alternative to fossil fuels due to reduction of cost and enhanced efficiency. However, the production performance of Solar PV over the period gets significantly impacted owing to a variety of problems such as dust, aging due to shading and soiling over the cell, hot spot, discoloration and corrosion for excessive atmospheric temperature, inadequate solar light, cell damage, and so on. In this research, a low-cost halogen-based artificial sun simulator is developed and deployed to examine the electrical properties of Solar PV in indoor conditions. Two monocrystalline and three polycrystalline PV panels under Standard Test Conditions, as well as a prototype 5 × 8 PV array, using this artificial light source, were evaluated rigorously for experimental purposes. With the help of a microcontroller-based I-V tracer and an actual data storage system, Open Circuit Voltage (Voc), Short Circuit Current (Isc), Maximum Power Voltage (Vmp), Maximum Power Current (Imp), and Maximum Power (Pmax) at three irradiance levels were measured and recorded. Utilizing Microsoft Excel software, the data logger's recorded data were analyzed and I-V and P-V curves were plotted. These data are extremely valuable for obtaining a good understanding of the validity of the Sun Simulator and the rate of deterioration of solar PV performance depending on irradiance. These data will aid the research community in future research regarding PV array performance monitoring, corresponding solution modeling, and developing cost-effective installation of large-scale PV arrays.

## Specifications Table

**Table:** 

Subject	Renewable Energy, Sustainability, and the Environment
Specificsubject area	Indoor testing of solar PV modules and array at STC and various irradiance levels utilizing an artificial sun simulator.
Type of data	Table, Figure.
How the datawere acquired	Experimental Investigation of PV modules and array in the laboratory.Testing Instruments: i) Halogen-based artificial Sun Simulator to generate artificial sunlight in the laboratory.Measuring Instruments: i) I-V tracer and an actual data storage system based on a microcontroller, ii) Light intensity measurement meter, Model: LX-1102, and iii) Temperature meter. Model: TA 298Software: Microsoft office software for data processing from the data logger.
Data format	Raw
Description ofdata collection	These data were acquired experimentally in the laboratory under Standard Test Conditions (STC) in an indoor environment.
Data sourcelocation	*Institution: Green University of BangladeshCity: DhakaCountry: Bangladesh*
Dataaccessibility	Repository name: Harvard DataverseData identification number (i.e. DOI number): 10.7910/DVN/0PVLDFDirect link to the dataset: https://dataverse.harvard.edu/dataset.xhtml?persistentId=doi:10.7910/DVN/0PVLDF

## Value of the Data

• The investigated data carried out the experimental authentication to validate the accuracy of the low-cost Artificial Sun simulator built-in lab (patent pending)*.*
• The experimental data and I-V curves represent the performance degradation of Monocrystalline and Polycrystalline PV modules at different levels of irradiance simulated in indoor conditions closely representing the outdoor temporal conditions.
• The I-V graphs are highly helpful to the industry and academic research communities in designing and producing efficient Solar PV systems for large-scale deployment and maintenance through rearrangement methods and replacements.
• PV-panel characteristics testing can be performed, and new PV modules can be tested compared to these baseline data capturing a real-life scenario created in a controlled indoor environment.

## 1 Data Description

**Fig. 1:**
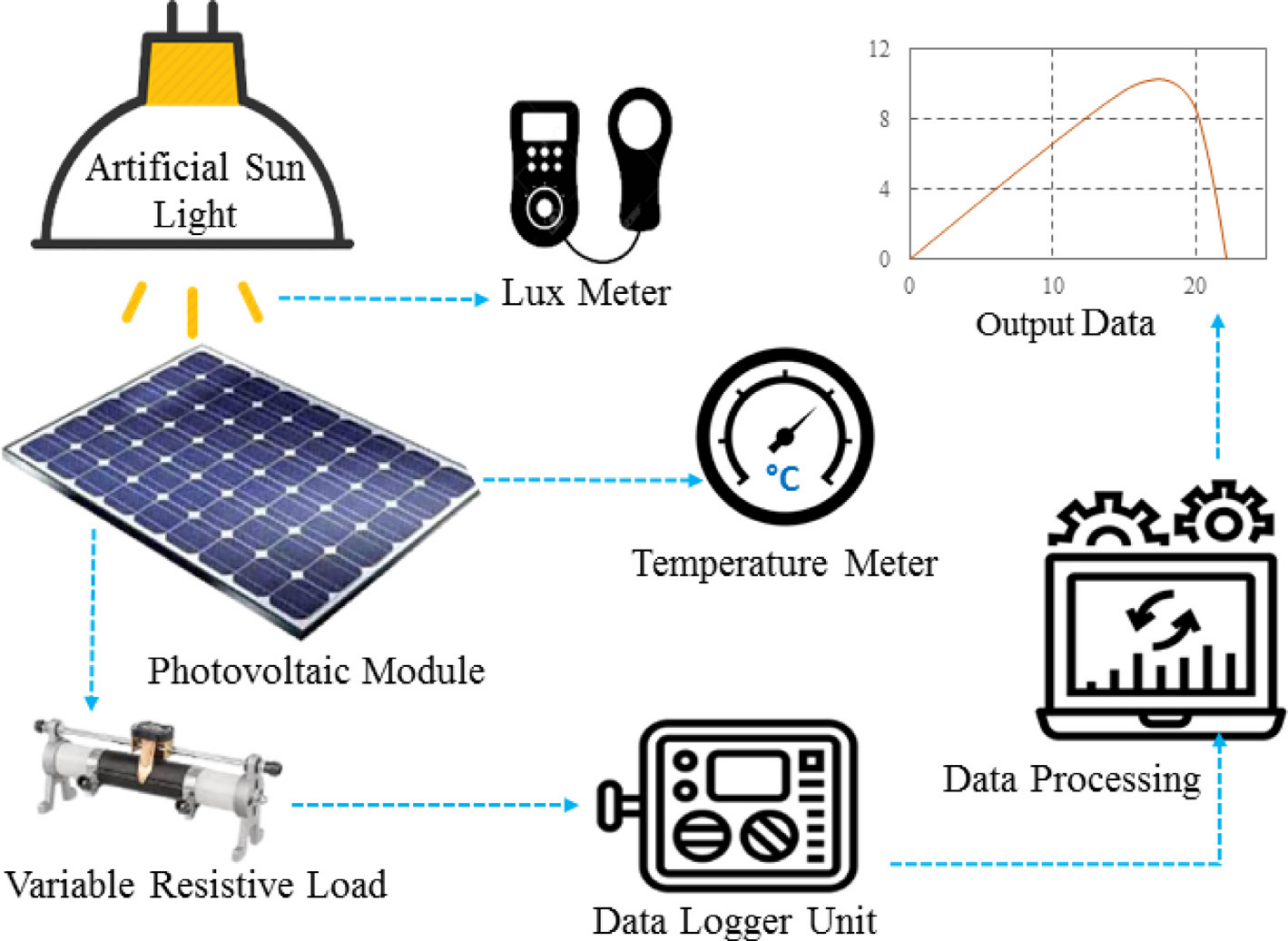
The schematic representation of the PV testing system's experimental setup.

The experimental dataset presented in this paper contains the electrical parameters of several PV modules and a prototype PV array that were examined in a laboratory setting using an artificial sun simulator [1] combined with microcontroller based I-V tracer and data-logger [2,3]. Fig. 1 depicts a schematic design of an experimental setup in an indoor environment. Tables 1 and 2 show the electrical characteristics of two monocrystalline PV modules, 10w monocrystalline panel 1 (MP1) and 10w monocrystalline panel 2 (MP2), while Tables 3 and 4 show the electrical characteristics of two polycrystalline PV panels, 10w polycrystalline panel 1 (PP1) and 10w polycrystalline panel 2 (PP2) at three irradiance level, 300 W/m^2^, 600 W/m^2^, and 1000 W/m^2^. The output parameters of a 20W polycrystalline panel 3 (PP3) are shown in Table 5. Table 6 shows the tested output parameters of a prototype 5 × 8 size PV array modules 1 (PAM1), where total 40 PV small modules (0.225W each) are connected in series-parallel (SP) configurations and the experimentation has been done at four different irradiance levels of 431 W/m^2^, 713 W/m^2^, 855 W/m^2^, and 1000 W/m^2^. Table 7 summarized the raw data which have been provided in the data sheets. Where, seven sheets are named as MP1, MP2, PP1, PP2, PP3, and PAM1 according to the panel and array name. In sheet MP1, MP2, PP1, PP2 and PP3 three test data are provided in each sheets considering the input light levels are 300 W/m^2^, 600 W/m^2^, and 1000 W/m^2^. While in sheet PAM1 the test data are given for irradiance levels of 431 W/m^2^, 713 W/m^2^, 855 W/m^2^, and 1000 W/m^2^. In each sheets the output variables are output voltage, current and power. Where the output power of PV panel and array is calculated using the following equations (1 and (2) respectively.

(1) $$P_{PV}=V_{PV}\times I_{PV}$$

(2) $$P_{Array}=V_{Array}\times I_{Array}$$

**Table 1:** Experimental observation of 10w monocrystalline panel 1 (MP1) at different irradiance levels

	Electrical Characteristic					
Irradiance Levels	Voc (V)	Isc (A)	Imp (A)	Vmp (V)	Pmax (W)	Panel size (cm^2^)
300 W/m^2^	22.29	0.11	0.10	17.58	1.76	702
600 W/m^2^	22.43	0.38	0.35	17.46	6.03	
1000 W/m^2^	22.47	0.63	0.59	17.08	10.10

**Table 2:** Experimental observation of 10w monocrystalline panel 2 (MP2) at different irradiance levels

	Electrical Characteristic					
Irradiance Levels	Voc (V)	Isc (A)	Imp (A)	Vmp (V)	Pmax (W)	Panel size (cm^2^)
300 W/m^2^	21.79	0.11	0.11	14.78	1.58	702
600 W/m^2^	21.85	0.38	0.35	15.49	5.77	
1000 W/m^2^	21.99	0.63	0.61	16.60	10.08

**Table 3:** Experimental observation of 10w polycrystalline panel 1 (PP1) at different irradiance levels

	Electrical Characteristic					
Irradiance Levels	Voc (V)	Isc (A)	Imp (A)	Vmp (V)	Pmax (W)	Panel size (cm^2^)
300 W/m^2^	22.43	0.11	0.9	18.66	1.77	750
600 W/m^2^	22.47	0.36	0.32	18.90	6.02	
1000 W/m^2^	22.55	0.6	0.53	18.99	10.07

**Table 4:** Experimental observation of 10w polycrystalline panel 2 (PP2) at different irradiance levels

	Electrical Characteristic					
Irradiance Levels	Voc (V)	Isc (A)	Imp (A)	Vmp (V)	Pmax (W)	Panel size (cm2)
300 W/m^2^	22.14	0.12	0.10	16.54	1.83	750
600 W/m^2^	22.18	0.40	0.35	17.16	6.11	
1000 W/m^2^	22.25	0.67	0.60	17.16	10.27

**Table 5:** Experimental observation of 20w polycrystalline panel 3 (PP3) PV panel at different irradiance levels

	Electrical Characteristic					
Irradiance Levels	Voc (V)	Isc (A)	Imp (A)	Vmp (V)	Pmax (W)	Panel size (cm^2^)
300 W/m^2^	21.19	0.24	0.21	16.76	3.55	1650
600 W/m^2^	21.18	0.79	0.72	16.48	11.86	
1000 W/m^2^	21.18	1.31	1.19	16.76	20.01

**Table 6:** Experimental observation of a 5 × 8 size PV array modules 1 (PAM1) at different irradiance levels

	Electrical Characteristic					
Irradiance Levels	Voc (V)	Isc (A)	Imp (A)	Vmp (V)	Pmax(W)	Array size
431 W/m^2^	18.00	0.37	0.32	12..6	3.89	5 × 8
713 W/m^2^	18.30	0.54	0.49	13.20	6.43	
855 W/m^2^	18.57	0.62	0.56	13.90	7.71	
1000 W/m^2^	18.85	0.74	0.67	13.47	9.02

**Table 7:** Summary of the raw data provided in the data sheets.

	Specifications		Data parameters	
Data sheet name	PV types	Ratting	Irradiance (W/m^2^)	Output variables
MP1	Monocrystalline	10W	1000, 600, 300	1. Module current, Ipv (A)2. Module voltage, Vpv (V)3. Module power, Ppv (W)
MP2	Monocrystalline	10W	1000, 600, 300	
PP1	Polycrystalline	10W	1000, 600, 300	
PP2	Polycrystalline	10W	1000, 600, 300	
PP3	Polycrystalline	20W	1000, 600, 300	
PAM1	Polycrystalline	9W	1000, 855, 713, 431	1. Array current, Iarray (A)2. Array voltage, Varray (V)3. Array power, Parray (W)

## 2 Experimental Design, Materials, and Methods

The experimental approach was carried out in the renewable energy lab at Green University of Bangladesh, for the measurement of electrical properties [4] of different PV modules from different manufacturer. The PV modules MP1, MP2, PP1, PP2, PP3 and array PAM1 are manufactured by different manufacturer. The instrumental arrangement for verifying a PV panel in an indoor environment utilizing artificial light is depicted in [5,6]. The artificial sun simulator frame is composed of steel and is laminated with color treatment for durability and safety purposes. Halogen based lights are used to produce artificial sun light. To control the input light intencity of the tested PV system an adjustable tray is employed to move the position of the PV module and array. In this case, four halogen lights (0.5KW each) are used to provide artificial light to the PV module, which is linked by aluminum bars. To minimize heat and speed up cooling, aluminum bars and two high-speed exhaust fans (3000 rpm) were employed. To sustain the Standard Test Condition (STC), a temperature meter and a lux meter are implemented in this experiment to measure the temperature and light intensity respectively. A micro-controller-based digital I-V tracer is used to determine the output power of the PV module, and a data logger is used to store the recorded values. A computer is assimilated to evaluate and analyse the measured data to demonstrate the electrical properties of the tested PV module.

A single test of a PV module must be performed in one minute and the temperature must not exceed 27°C at the indoor experimentation, as per European standards. To maintain the temperature at 25°C, the entire room was under the surveillance of the air conditioner. Besides, there was an additional high-speed cooling fan to retain the PV panel cool. In this work, the data has been taken according to STC, and to ensure the highest power output, the same test has been performed five times for each PV module. By adjusting the position of the movable tray, the modules were tested at three different irradiance levels: full sun, medium sun, and low sun, with irradiances of 300 W/m^2^, 600 W/m^2,^ and 1000 W/m^2^.

## CRediT Author Statement

**Ahmed Al Mansur:** Conceptualization, Methodology, Writing an original draft, Data curation, Validation, Formal analysis, Investigation; **Md. Imamul Islam:** Conceptualization, Methodology, Writing an original draft, Software, Formal analysis, Data curation, and Investigation; **Mohammad Kamrozzaman Kiron:** Writing – original draft, Investigation, and Data Curation; **Mohammad Asif ul Haq:** Investigation, Data curation, review & editing; **Md. Hasan Maruf:** Investigation and Data curation; **Ratil Hasnat Ashique:** Investigation, Data curation, review & editing; **ASM Shihavuddin:** Supervision, Project Administration, Investigation, and Data curation; **Md. Ruhul Amin:** Conceptualization, Data curation, and Investigation.

## Declaration of Competing interest

The authors declare that they have no known competing financial interests or personal relationships that could have appeared to influence the work reported in this paper.

## Data Availability

Experimental Dataset of of PV Modules in Indoor Environment using Artificial Sun Simulator (Original data) (Dataverse).

## Appendix Supplementary materials

Image, application 1

Image, application 2
