# Supplementary material for: Electrical experimental data collection of polycrystalline and monocrystalline photovoltaic modules in an indoor environment using artificial sun simulator
Source: Data Brief. 2022 Jun 14;43:108389. doi: 10.1016/j.dib.2022.108389 (PMC9249606; doi:10.1016/j.dib.2022.108389)
Supplement: Supplementary file 1 [file mmc1.pdf]

# Investigation of PV Modules Electrical Characteristics for Laboratory Experiments using Halogen Solar Simulator

Ahmed Al Mansur*, Md. Imamul Islam*, Mohammad Asif ul Haq*, Md. Hasan Maruf*, ASM Shihavuddin*, and Md. Ruhul Amin†
Department of Electrical and Electronic Engineering,Green University of Bangladesh, Dhaka, Bangladesh*
Department of Electrical and Electronic Engineering, Islamic University of Technology, Gazipur, Dhaka, Bangladesh†
Email: mansur@eee.green.edu.bd, imamulir2382@gmail.com, asiful@eee.green.edu.bd, maruf@eee.green.edu.bd, shihav@eee.green.edu.bd, and ruhul@iut-dhaka.edu

2020 2nd International Conference on Sustainable Technologies for Industry 4.0 (STI) |978-1-6654-0489-1/20/$31.00 ©2020 IEEE | DOI: 10.1109/STI50764.2020.9350496

***Abstract*— The electrical characteristics of the photovoltaic (PV) module degrades due to several factors such as non-uniform aging, high atmospheric temperature, insufficient sun lights, shading, dust, soiling, cell damage, etc. In order to investigate the electrical performance of PV modules at such conditions, indoor solar simulators are commonly used for experimental research. In this work, a solar simulator is developed for a university lab to test the electrical characteristics of PV modules and array for low-cost experimental purposes. A microcontroller-based current-voltage (I-V) tracer with a real-time data logger system is used to maintain the reliability of the measuring system. The experimental investigation of the halogen-based solar simulator is carried out using both polycrystalline and monocrystalline PV panels of different power ratings. A prototype (5×8) PV array with 40 PV cells is tested by the solar simulator at a different level of irradiance. The experimental result shows that the average measurement error is ±0.022% which is within the acceptable range of a commercial simulator.**

***Keywords*— *Photovoltaic module and array, PV electrical characteristics, degradation, solar simulator.***

## I. INTRODUCTION

The development of energy is played a key role in the economic growth of any country [1]. However, most of the energy is generated from non-renewable energy sources (NRES) [2], like fossil fuels, coal, oil, gas, which causes environmental pollution, and NRES were arranged once in millions of years [3]. Hance, to reduce ecological pollution and uncertainty of NRES led an alternative solution as the renewable energy sources (RES) [4], such as photovoltaic, wind [5], ocean wave, biogas. The photovoltaic energy is one of the major available RES all over the world. Nevertheless, the weakness of the PV system is its low conversion efficiency [6], high operation cost, power degradation due to aging, shading, dust, soiling, etc. [7-10]. Therefore, a significant number of research and developments are carried out by the researcher to advance the total efficiency of the PV arrangement. Hence, the PV module or array testing is frequently required in the renewable energy laboratory or photovoltaic laboratory for experimental investigation purposes using solar simulator.

The work in [11], a solar simulator is developed using halogen light to test PV module, but the test panel size is only 10×10 cm$^2$. In [12], a halogen-based solar simulator is developed with a test panel size of 24×100 cm$^2$, without any commercial I-V tracer. In [13], a solar simulator is designed for renewable energy laboratory to test PV module electrical characteristics using xenon, halogen, and LED bulbs, without any hardware implementation. The work in [14], a halogen-based solar simulator is used to test the PV module for laboratory purpose. Moreover, for PV thermal testing purposes halogen-based solar simulator is used in the renewable energy laboratory [15]. Therefore, it is evident that a halogen-based solar simulator is very effective to test the PV module at an indoor laboratory testing system.

The testing of PV modules using an indoor artificial sunlight simulator provides fast results with easy operation and standard test environment to control the testing processes [16]. However, at outdoor with uncontrollable weather condition it is very difficult to test a PV module, while the irradiance level of sun light is changing continuously. Hance, laboratory-based PV testing system is recommended by the IEC standard [17-20]. The performance of PV array modules degrades due to different mismatch conditions causes by nonuniform aging, shading, soiling, dust, etc. While advanced MPPT converter and inverter are desired to extract maximum power output. Hence, to develop such advanced devices for the PV system, an experimental investigation is needed to be done at the laboratory using constant irradiance [21-23]. Therefore, the design and implementation of a fast and reliable solar simulator for laboratory experimentation is the major aim of this research paper.

According to the European Commission, Joint Research Centre (JRC) report [24], EUR 24359 EN, the measurement of electrical characteristics of the commercial PV module is acceptable using an indoor solar simulator. However, a single test must be done within one-minute time at recommended test environment with 25°C temperature, light at 1000 W/m$^2$, and AM 1.5G according to IEC standard [25]. Therefore, in this work, a microcontroller-based fast and smart I-V tracking system is used to evaluate the I-V characteristics of both PV modules and array by maintaining the STC. Besides, a digital data logger is used for continuous data acquisition purposes. Another important factor of the solar simulator is the uniformity of light over a focal plane and matching of the light spectrum with sunlight. The proposed halogen-based solar simulator

978-1-6654-0489-1/20/$31.00©20XX IEEE

Authorized licensed use limited to: Univ of Calif Santa Barbara. Downloaded on May 21,2021 at 06:45:32 UTC from IEEE Xplore. Restrictions apply.

is designed considering these factors. Moreover, some polycrystalline and monocrystalline PV panels are tested using the proposed indoor PV experimental setup to validate the effectiveness of the solar simulator.

## II. Electrical Characteristics of PV Module

A PV panel is a current source, where the output current is proportional to the input light level. The equivalent circuit of a PV cell or panel is exposed in Fig. 1. The output power of a PV module is proportion to its short circuit current. Therefore, the output power is correlated on the incident light directly [26]. Hence, for indoor PV testing purposes, a halogen-based solar simulator is effective due to the spectral matching with daylight [27]. The electrical characteristics of the PV panel are shown in Fig. 2. Where current-voltage (I-V) and power-voltage (P-V) curves are showing the nonlinear characteristics of a PV module. The maximum power, Pmax, can be calculated by using any of the following equations.

$$P_{max} = V_{oc} * I_{SC} * FF \quad (1)$$

$$P_{max} = \eta * G * A \quad (2)$$

$$P_{max} = I_{mp} * V_{mp} \quad (3)$$

Where, $V_{oc}$ = Open circuit voltage, $I_{SC}$ = Short circuit current, FF = Fill factor, η = Efficiency, A = Area of the panel, G = Irradiance (W/m$^2$), $I_{mp}$= Max power point current, $V_{mp}$ = maximum power point voltage. The fill factor and efficiency of a PV panel is calculated in this work by using the equation (4) and (5) respectively.

$$FF = \frac{I_{mp} * V_{mp}}{V_{OC} * I_{SC}} \quad (4)$$

$$\eta = \frac{I_{mp} * V_{mp}}{G * A} \times 100\% \quad (5)$$

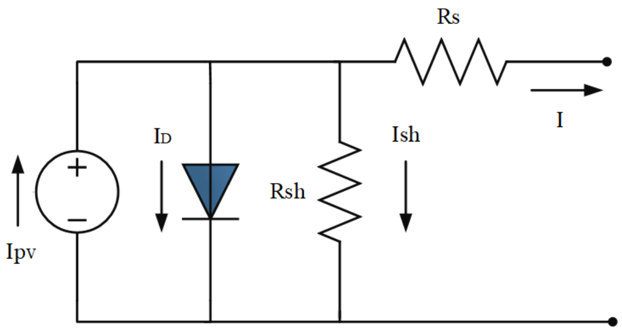


Fig. 1. The electrical equivalent circuit of a photovoltaic module.

### A. *Polycrystalline PV Modules*

Here, three polycrystalline PV panles are used to investigate their electrical characteristics using the proposed halogen-based solar simulator. The rated electrical specifications of the three polycrystalline modules, Poly 1, Poly 2, and Poly 3 are summarized in Table I. The power rating of Poly 1, Poly 2, and Poly 3 modules are 10.175 W, 10.063 W, and 22.692 W respectively.

TABLE I. Specifications of Polycrystalline PV Panels

| Electrical Specification | 10W Poly 1 | 10W Poly 2 | 20W Poly 3 |
|---|---|---|---|
| Open circuit voltage, *Voc (V)* | 22.17 | 21.16 | 22.43 |
| Short circuit current, *Isc (A)* | 0.60 | 0.67 | 1.31 |
| Max power current, *Imp (A)* | 0.56 | 0.58 | 1.22 |
| Max power voltage, *Vmp (V)* | 18.17 | 17.35 | 18.6 |
| Area of the module, A (cm$^2$) | 750 | 750 | 1650 |
| Max power at MPP, *Pmax (W)* | **10.175** | **10.063** | **22.692** |

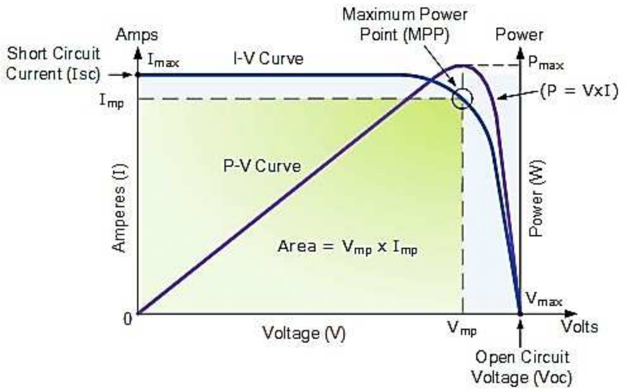


Fig. 2. The electrical characteristics of a photovoltaic panel.

### B. *Monocrystalline PV Modules*

Two monocrystalline PV modules are tested by the artificial sun simulator. Their rated electrical characteristics are tabulated in Table II. The rated power of the monocrystalline modules, Mono 1 and Mono 2 are 10.157 W and 10.08 W respectively.

TABLE II. Specifications of Monocrystalline PV Panels

| Electrical Specification | 10W Mono 1 | 10W Mono 2 |
|---|---|---|
| Open circuit voltage, *Voc (V)* | 21.96 | 21.8 |
| Short circuit current, *Isc (A)* | 0.63 | 0.63 |
| Max power current, *Imp (A)* | 0.57 | 0.56 |
| Max power voltage, *Vmp (V)* | 17.82 | 18.0 |
| Area of the module, A (cm$^2$) | 702 | 702 |
| Max power at MPP, *Pmax (W)* | **10.157** | **10.08** |

## III. Experimental Work

The technique of experimentation is demonstrated here to measure the electrical characteristics of different power rated polycrystalline and monocrystalline PV panels and a prototype 5×8 PV array. The experimental process of testing a PV module at the laboratory using artificial sunlight is exposed in Fig.3. Wherever the commercial halogen lights are used as an alternate of sunlight to provide input power to the PV module. The halogen lights are powered by 220V AC power supply. To maintain the STC (25°C, 1000 W/m$^2$) temperature meter and lux meter are used here. The output power of the PV module is measured by a digital I-V tracer and a datalogger is utilized to save the measured data. A computer is incorporated to analyze and process the measured data to illustrate the

Authorized licensed use limited to: Univ of Calif Santa Barbara. Downloaded on May 21,2021 at 06:45:32 UTC from IEEE Xplore. Restrictions apply.

electrical characteristics of the experimented PV module.

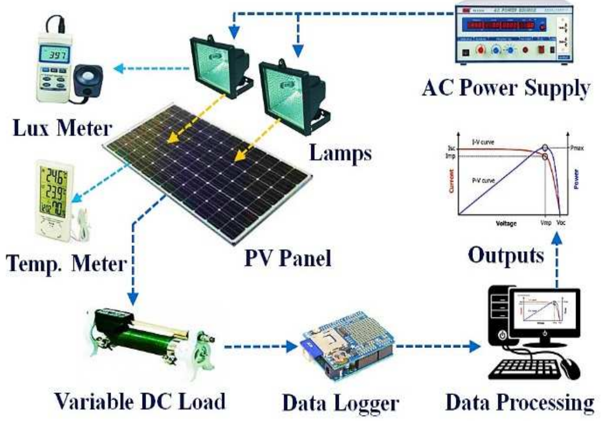


Fig. 3. Schematic diagram of the experimental setup of the PV testing system.

The proposed halogen-based solar simulator is described in the following subsection. The experimental setup of the halogen-based solar simulator for testing the PV module is shown in Fig. 4. The dimension of the sun simulator is (107 cm × 92 cm × 72 cm). It is made of steel frame and laminated by color coating. A moveable tray is used to place the PV module parallel with the halogen lights. For this work, four halogen lights (0.5kW each) are used to test 10W PV modules and the lights are connected with aluminum bars. The aluminum bars are used to enhance the cooling system by reducing the temperature of halogen lights. However, the experimentation is done according to the IEC standard by maintain the room temperature at 25ºC [18] using an efficient air conditioner. Besides, the high-speed exhaust fans (3000 rpm) are placed on the top of the simulator frame to provide faster cooling during the experimentation. Besides a smart cooling fan with controllable speed is placed in between the lights and the PV module to adjust the module temperature. According to the European standard [22], at the indoor test condition of the PV module, a single test should be done within one minute, and temperature must not exceed 27˚C during the experiment. Therefore, in this work, the experiment is done by maintaining the test conditions.

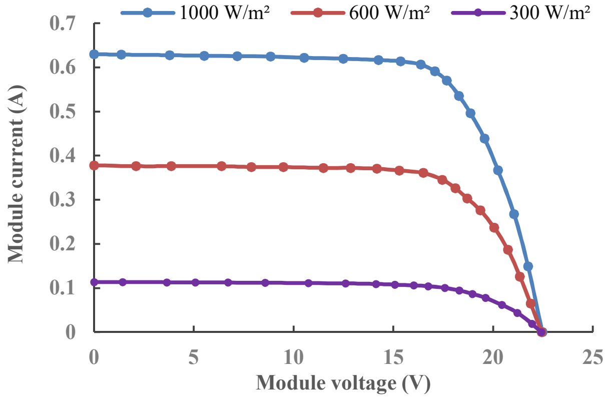


Fig. 4. Voltage-current characteristics of 10W monocrystalline module 1 obtained at three different irradiance level

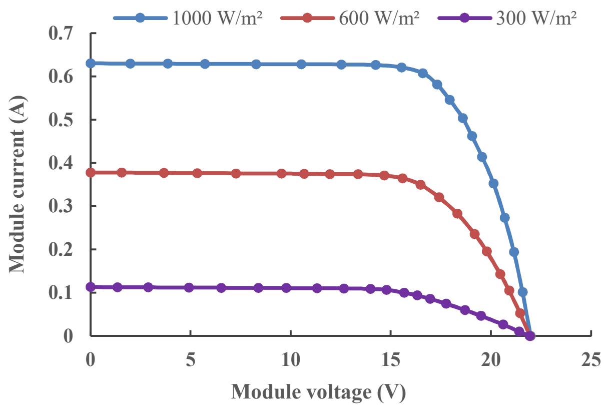


Fig. 5. Voltage-current characteristics of 10W monocrystalline module 2 obtained at three different irradiance level

The measurement of light intensity and the temperature is completed by using two meters, LX-1102 and TA298 respectively. The electrical characteristics of the PV module are measured and recorded continuously by the microcontroller-based I-V tracer by varying the resistive load. The light intensity of the solar simulator is varied at different levels by moving the test tray accordingly. In this work, the individual test is redid five times for each PV module to acquire the maximum output power. Besides, five minutes of the time interval is provided between two consequent tests to ensure the module temperature is at 25ºC. Therefore, after a daylong experimental investigation, the output characteristics are measured and recorded for three polycrystalline panels, two monocrystalline panels, and a 5×8 PV array.

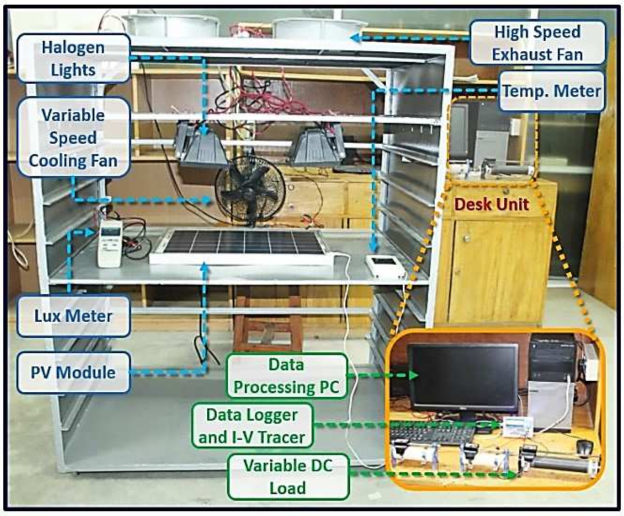


Fig. 6. Experimental setup of the halogen based solar simulator for PV module testing system.

## IV. RESULTS AND DISCUSSION

The outputs from the experimentations are investigated here. The electrical characteristics, I-V curves, of two monocrystalline PV modules, Mono 1 and Mono 2 are illustrated in Fig. 5 and Fig. 6 respectively. The modules are tested at three different irradiance levels such as full sun, medium sun and low sun and the irradiance are 1000 $Wm^{-2}$, 600 $Wm^{-2}$ and 300 $Wm^{-2}$ respectively. Similarly, the

Authorized licensed use limited to: Univ of Calif Santa Barbara. Downloaded on May 21,2021 at 06:45:32 UTC from IEEE Xplore. Restrictions apply.

output characteristics of three polycrystalline PV modules, Poly 1, Poly 2, and Poly 3 are illustrated in Fig. 7, Fig. 8, and Fig. 9 respectively. The proposed solar simulator has provided three different levels of irradiance to obtain the output characteristics of both polycrystalline and monocrystalline PV modules successfully.

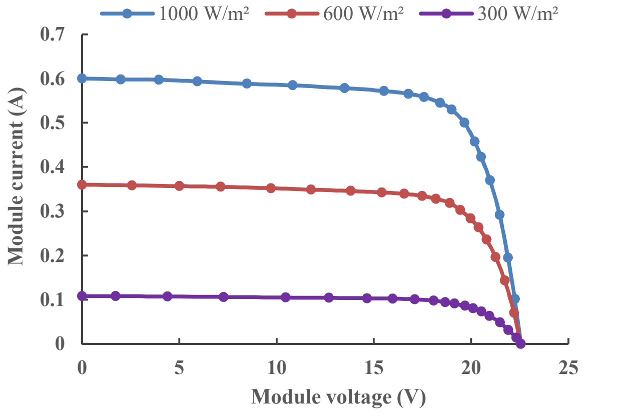


Fig. 7. Voltage-current characteristics of 10W polycrystalline module 1 obtained at three different irradiance level

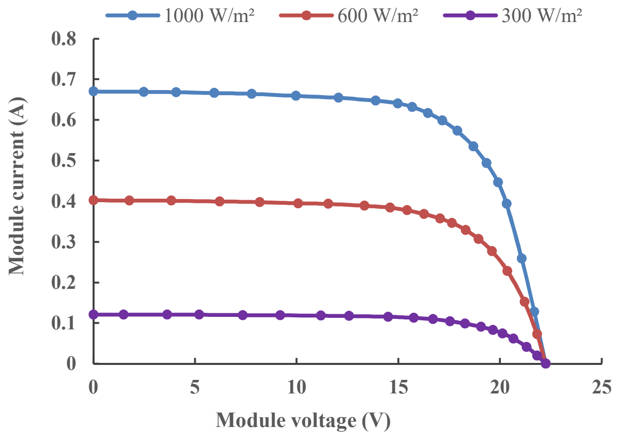


Fig. 8. Voltage-current characteristics of 10W polycrystalline module 2 obtained at three different irradiance level

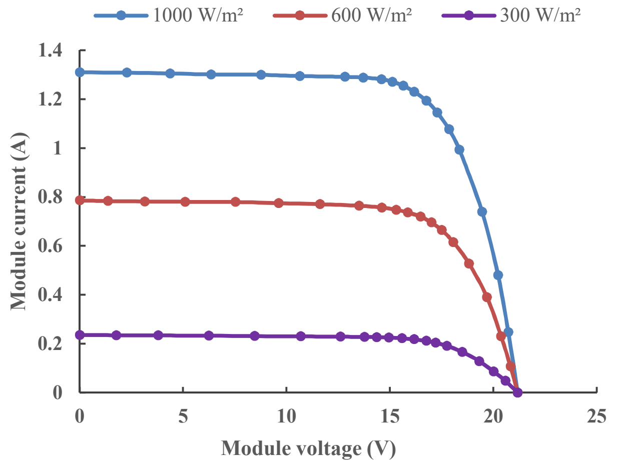


Fig. 9. Voltage-current characteristics of 20W polycrystalline module 3 obtained at three different irradiance levels.

The tested results are analyzed for output power and efficiency of the modules. The results are summarized in Table III. Where, the module size, output power, and efficiency are tabulated for five different PV modules. The size of the two monocrystalline PV modules is the same and the area is 702 $cm^2$. Whereas both the Poly 1 and Poly 2 modules size are 750 $cm^2$ but Poly 3 is different in power ratting and its size is 1650 $cm^2$. The efficiency of Mono1 and Mono 2 modules is 14.469 % and 14.358 % respectively. While the polycrystalline module is performed lower efficiency than monocrystalline modules. The result shows that Poly 1, Poly 2, and Poly 3 are achieved an efficiency of 13.566 %, 13.417 %, and 13.752 % respectively.

TABLE III. EFFICIENCY CALCULATED FOR DIFFERENT PV MODULES

| PV module | Size ($cm^2$) | Output Power (W) | Efficiency (%) |
|---|---|---|---|
| 10W Mono 1 | 702 | 10.159 | **14.469** |
| 10W Mono 2 | 702 | 10.082 | 14.358 |
| 10W Poly 1 | 750 | 10.178 | 13.566 |
| 10W Poly 2 | 750 | 10.066 | 13.417 |
| 20W Poly 3 | 1650 | 22.695 | 13.752 |

However, the efficiency of Poly 3 is higher than both the Poly 1 and Poly 2 modules. The variation of the percentage of efficiency among the monocrystalline and the polycrystalline PV modules is depicted in Fig. 10. The literature shows that the mono-crystalline modules are better than polycrystalline modules concerning efficiency. Therefore, according to the experimental results, the proposed experimental testing system of the PV module using a halogen based solar simulator is performed successfully.

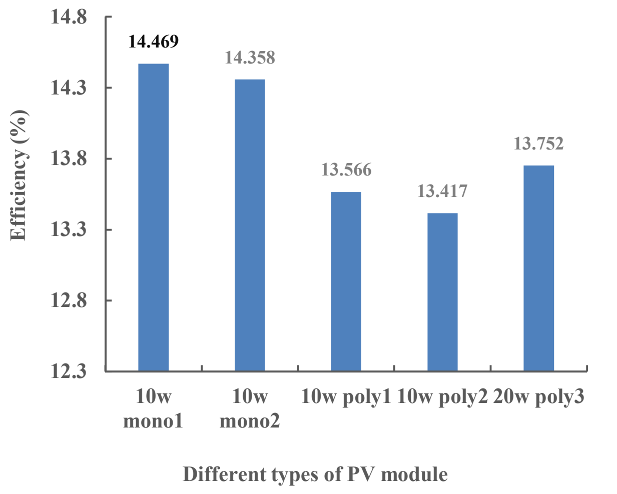


Fig. 10. Efficiency calculated from the experimental data for five different PV modules using the solar simulator.

In this subsection, the accuracy of the proposed solar simulator-based PV module testing system has been investigated by calculating the measurement error in output power. The percentage of measurement error, ($\%E_m$) is calculated by applying the subsequent equation.

Authorized licensed use limited to: Univ of Calif Santa Barbara. Downloaded on May 21,2021 at 06:45:32 UTC from IEEE Xplore. Restrictions apply.

$$\%E_m = \left| \frac{P_{stc}^{mpp} - P_{exp}^{mpp}}{P_{stc}^{mpp}} \right| \times 100 \qquad (6)$$

Wherever $P_{stc}^{mpp}$ and $P_{exp}^{mpp}$ are represented as the maximum power of the PV module at rated (STC) condition and experimentally measured condition. The $\%E_m$ for five different PV modules is illustrated in Fig. 11. Where the minimum value of $\%E_m$ is 0.0132 % obtained for 20 W polycrystalline module and the maximum $\%E_m$ is 0.0298% achieved for Poly 2 module. The average $\%E_m$ is calculated for the five examined modules and the value is 0.022%. The error value is quite low, which is another significant parameter of the proposed system.

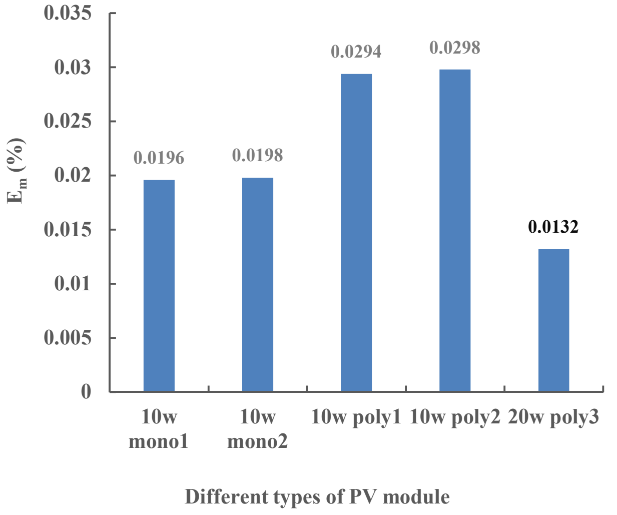


Fig. 11. Measurement error calculated from the experimental output power of five different PV modules using the solar simulator.

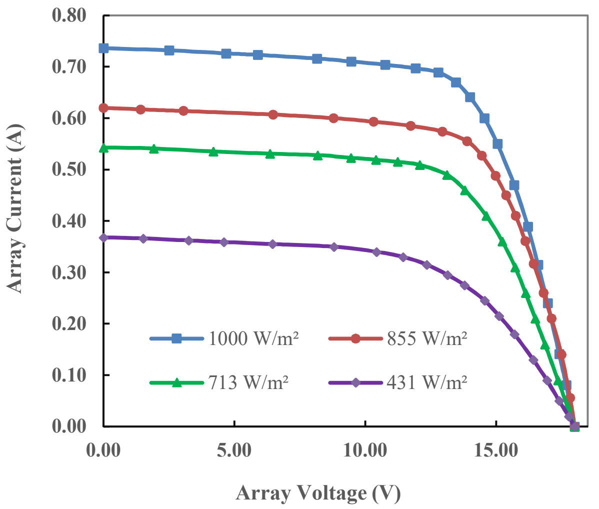


Fig. 12. Voltage-Current characteristics of 9W PV array obtained at four different irradiance levels of the solar simulator.

In this subsection, the experimental results are analyzed for a 9 W, 5×8 PV array. In Fig. 12 characteristics curves are depicted for the array using four different irradiance levels of the solar simulator and the irradiances are 1000 $Wm^{-2}$, 855 $Wm^{-2}$, 713 $Wm^{-2}$, and 431 $Wm^{-2}$ respectively. The P-V curves are illustrated in Fig. 13 for the same irradiance levels. The array output characteristics are obtained successfully. This type of small array can be used for the experimental investigation of mismatch power loss (MPL) due to shading, dust, soiling, nonuniform aging effects of PV modules in the array. Therefore, the proposed solar simulator will be effective in the advanced research, in the future, using the 5×8 proto-type PV array.

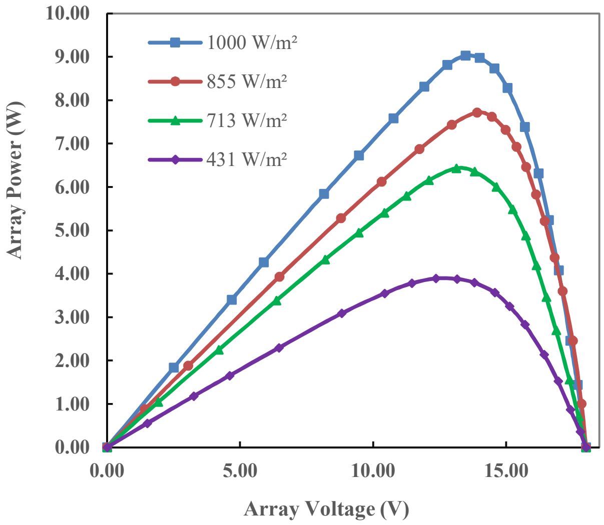


Fig. 13. Voltage-power characteristics of 9W PV array obtained at four different irradiance level of solar simulator.

## V. CONCLUSION

In this investigation, evaluation and experimental performance of the halogen solar simulator for the PV module and array testing system are presented. The experimental analysis is done by using polycrystalline and monocrystalline modules with different irradiance conditions. The core results accomplished by this paper are specified here:

- For the validation of the accuracy of the planned solar simulator, experimental authentication is carried out. The examinations prove that the percentage of errors is in a satisfactory range.
- The average measurement error is achieved by 0.022% experimentally.
- The maximum amount of the electrical efficiency of the monocrystalline module is achieved as 14.469 % and similarly, for the polycrystalline module, electric efficiency is achieved as 13.752 % at 1000 $W/m^2$ experimentally.
- Furthermore, this work will contribute to PV array performance investigations in the future.

## VI. ACKNOWLEDGMENT

The authors would like to acknowledge the laboratory support of the Department of Electrical and Electronic Engineering (EEE) at the Green University of Bangladesh. Extraordinary thanks to ReSES Center for creating a collaborative research platform for this work.

Authorized licensed use limited to: Univ of Calif Santa Barbara. Downloaded on May 21,2021 at 06:45:32 UTC from IEEE Xplore. Restrictions apply.

REFERENCES

[1] Fayaz, H., Nasrudin Abd Rahim, Md Hasanuzzaman, Ahmad Rivai, and Rehena Nasrin. "Numerical and outdoor real time experimental investigation of performance of PCM based PVT system." Solar Energy 179 (2019): 135-150. S.

[2] Md Hasan Maruf, Mohammad Asif ul Haq Suman Kumar Dey, Ahmed Al Mansur, A.S.M.Shihavuddin " Adaptation for sustainable implementation of Smart Grid in developing countries like Bangladesh." Energy Reports (2020).

[3] M. A. ul Haq, A. Islam, A. Shihavuddin, M. H. Maruf, A. Al Mansur, and M. Y. Hassan, "Enhanced Energy Savings in Indoor Environments with Effective Daylight Utilization and Area Segregation," Symmetry, vol. 12, no. 8, p. 1313, Aug. 2020.

[4] Shihavuddin, A. S. M., Xiao Chen, Vladimir Fedorov, Anders Nymark Christensen, Nicolai Andre Brogaard Riis, Kim Branner, Anders Bjorholm Dahl, and Rasmus Reinhold Paulsen. "Wind Turbine Surface Damage Detection by Deep Learning Aided Drone Inspection Analysis." Energies 12, no. 4 (2019): 676.

[5] Shihavuddin, A. S. M., Xiao Chen, Vladimir Fedorov, Anders Nymark Christensen, Nicolai Andre, Brogaard Riis, Kim Branner, Anders Bjorholm Dahl, and Rasmus Reinhold Paulsen. "Wind turbine maintenance cost reduction by deep learning aided drone inspection analysis." (2019).

[6] A. A. Mansur, M. Amin, and K. K. Islam, "Performance Comparison of Mismatch Power Loss Minimization Techniques in Series-Parallel PV Array Configurations," *Energies,* vol. 12, p. 874, 2019.

[7] Ahmed Al Mansur, Md Ruhul Amin, Kazi Khairul Islam, "Comparative Analysis of Mismatch Power Loss Reduction Techniques for Photovoltaic Series Parallel Array Configurations"- IAPE '19, Oxford, United Kingdom ISBN: 978-1-912532-05-6, March, 2019.

[8] Kalogirou, Soteris A., Rafaela A. Agathokleous, and Gregoris Panayiotou. "Indoor testing of solar systems: a solar simulator for multidisciplinary research on solar technologies." Chem Eng Trans 39 (2014).

[9] Esen, Vedat, Şafak Sağlam, and Bülent Oral. "Light sources of solar simulators for photovoltaic devices: A review." Renewable and Sustainable Energy Reviews 77 (2017): 1240-1250.

[10] Yandri, Erkata. "Uniformity characteristic and calibration of simple low-cost compact halogen solar simulator for indoor experiments." International Journal of Low-Carbon Technologies 13, no. 3 (2018): 218-230.

[11] Grandi, Gabriele, Anastasiia Ienina, and Marinel Bardhi. "Effective low-cost hybrid LED-halogen solar simulator." IEEE Transactions on Industry Applications 50, no. 5 (2014): 3055-3064.

[12] Samiran Roy, Md. Rashedul Islam, Parimol Kumar Sarkar, Md. Ashikul Haque, Liton Kumar Biswas, 2014, Study the Characteristics of PV Module Using a Sun Simulator, INTERNATIONAL JOURNAL OF ENGINEERING RESEARCH & TECHNOLOGY (IJERT) Volume 03, Issue 04 (April 2014).

[13] Dafalla, Yousif, and Mohamed Osman. "A solar simulator for the Renewable Energy instruction laboratory." In 2016 IEEE Conference on Technologies for Sustainability (SusTech), pp. 235-239. IEEE, 2016.

[14] Nema, R. K., Savita Nema, and Gayatri Agnihotri. "Computer simulation-based study of photovoltaic cells/modules and their experimental verification." International Journal of Recent Trends in Engineering 1, no. 3 (2009): 151-156.

[15] Saxena, Ashish, Sandip Deshmukh, Somanath Nirali, and Saurabh Wani. "Laboratory based experimental investigation of photovoltaic (PV) thermo-control with water and its proposed real-time implementation." Renewable Energy 115 (2018): 128-138.

[16] Parupudi, Ranga Vihari, Harjit Singh, and Maria Kolokotroni. "Sun Simulator for Indoor Performance assessment of Solar Photovoltaic Cells." Energy Procedia 161 (2019): 376-384.

[17] A. Al Mansur, S. Ferdous, Z. B. Shams, M. R. Islam, M. Rokonuzzaman, and M. A. Hoque, "An experimental investigation of the real time electrical characteristics of a PV panel for different atmospheric conditions in Islamic University of Technology (OIC), Gazipur, Bangladesh," in *Utility Exhibition on Power and Energy Systems: Issues &* Prospects for Asia (ICUE), 2011 International Conference *and*, 2011, pp. 1-8.

[18] Muhammad Towhidur Rahman, Ahmed Al Mansur, Nahyan Al Mahmud, Mahmoodul Islam, Taskin Jamal, "Development of Electrical Behavioral Model of an Arbitrary Solar Cell to Amend the PSPICE Simulation Performance", IEEE, International Conference and Utility Exhibition, 28-30 September 2011

[19] A. A. Mansur, M. R. Amin and K. K. Islam, "Determination of Module Rearrangement Techniques for Non-uniformly Aged PV Arrays with SP, TCT, BL and HC Configurations for Maximum Power Output," 2019 International Conference on Electrical, Computer and Communication Engineering (ECCE), Cox's Bazar, Bangladesh, 2019, pp. 1-5.

[20] Tausif Ali, Ahmed Al Mansur, Zubaeer Bin Shams, S.M. Ferdous, Md. Ashraful Hoque, "An Overview of Smart Grid Technology in Bangladesh: Development and Opportunities", IEEE, International Conference and Utility Exhibition, 28-30 September 2011, AIT, Pattaya City, Thailand, Ref. No: S 02.2.

[21] A. Al Mansur and M. R. Amin, "Performance Investigation of Different PV Array Configurations at Partial Shading Condition for Maximum Power Output," *2019 International Conference on Sustainable Technologies for Industry 4.0 (STI)*, Dhaka, Bangladesh, 2019, pp. 1-5.

[22] Ahmed Al Mansur, Biplob Shil, Kazi Khairul Islam, Md Ashraful Hoque, Muhibul Haque Bhuyan "Design and Implementation of a Solar Radiation Meter using PV panel as a Sensor" –Green University Journal, Bangladesh, Volume 1, Number 1, July 2014.

[23] Ahmed Al Mansur, Kazi Khairul Islam, "Combined Constant Voltage and Perturb and Observe Method Based Algorithm for Fast and Efficient Maximum Power Point Tracking Using Buck-Boost Converter" Green University Journal, Volume 4, Number 1-2, December 2013.

[24] Dunlop, Ewan D., Fernando Fabero, Gabi Friesen, Werner Herrmann, J. Hohl-Ebinger, Hans-Dieter Mohring, Harald Müllejans et al. "Guidelines for PV power measurement in industry." (2010).

[25] P. Devices—Part, "Measurement of Photovoltaic Current-Voltage Characteristics," *CEI/IEC,* pp. 60904-1.

[26] I. Standard, "60891. Photovoltaic Devices. Procedures for Temperature and Irradiance Corrections to Measured IV Characteristics," *International Electrotechnical Commission,* 2009.

[27] Peter Michael, "A Conversion Guide: Solar Irradiance and Lux Illuminance ", IEEE Dataport, 2019. [Online]. Available: http://dx.doi.org/10.21227/mxr7-p365. Accessed: Jun. 01, 2020.


Authorized licensed use limited to: Univ of Calif Santa Barbara. Downloaded on May 21,2021 at 06:45:32 UTC from IEEE Xplore. Restrictions apply.
